# The Effect of Rifampicin on Darunavir, Ritonavir, and Dolutegravir Exposure within Peripheral Blood Mononuclear Cells: a Dose Escalation Study

**DOI:** 10.1128/aac.00136-22

**Published:** 2022-05-18

**Authors:** Amedeo De Nicolò, Andrea Calcagno, Ilaria Motta, Elisa De Vivo, Antonio D’Avolio, Giovanni Di Perri, Lubbe Wiesner, Isma-eel Ebrahim, Gary Maartens, Catherine Orrell, Helen McIlleron

**Affiliations:** a Laboratory of Clinical Pharmacology and Pharmacogenetics, Department of Medical Sciences, University of Turin, Turin, Italy; b Unit of Infectious Diseases, Department of Medical Sciences, University of Turin, Turin, Italy; c Division of Clinical Pharmacology, Department of Medicine, University of Cape Town, Cape Town, South Africa; d Wellcome Centre for Infectious Diseases Research in Africa (CIDRI-Africa), Institute of Infectious Disease and Molecular Medicine, University of Cape Town, Cape Town, South Africa; e Desmond Tutu HIV Centre, Institute of Infectious Diseases and Molecular Medicine (IDM) and Department of Medicine, University of Cape Town, Cape Town, South Africa

**Keywords:** darunavir, rifampicin, dolutegravir, drug-drug interaction, PBMC, pharmacokinetics, drug interactions

## Abstract

Ritonavir-boosted darunavir (DRV/r) and dolutegravir (DTG) are affected by induction of metabolizing enzymes and efflux transporters caused by rifampicin (RIF). This complicates the treatment of people living with HIV (PLWH) diagnosed with tuberculosis. Recent data showed that doubling DRV/r dose did not compensate for this effect, and hepatic safety was unsatisfactory. We aimed to evaluate the pharmacokinetics of DRV, ritonavir (RTV), and DTG in the presence and absence of RIF in peripheral blood mononuclear cells (PBMCs). PLWH were enrolled in a dose-escalation crossover study with 6 treatment periods of 7 days. Participants started with DRV/r 800/100 mg once daily (QD), RIF and DTG were added before the RTV dose was doubled, and then they received DRV/r 800/100 twice daily (BD) and then 1,600/200 QD or vice versa. Finally, RIF was withdrawn. Plasma and intra-PBMC drug concentrations were measured through validated liquid chromatography-tandem mass spectrometry (LC-MS/MS) methods. Seventeen participants were enrolled but only 4 completed all study phases due to high incidence of liver toxicity. Intra-PBMC DRV trough serum concentration (*C*_trough_) after the addition of RIF dropped from a median (interquartile range [IQR]) starting value of 261 ng/mL (158 to 577) to 112 ng/mL (18 to 820) and 31 ng/mL (12 to 331) for 800/100 BD and 1,600/200 QD DRV/r doses, respectively. The DRV intra-PBMC/plasma ratio increased significantly (*P* = 0.003). DTG and RIF intra-PBMC concentrations were in accordance with previous reports in the absence of RIF or DRV/r. This study showed a differential impact of enzyme and/or transporter induction on DRV/r concentrations in plasma and PBMCs, highlighting the usefulness of studying intra-PBMC pharmacokinetics with drug-drug interactions. (This study has been registered at ClinicalTrials.gov under registration no. NCT03892161.)

## INTRODUCTION

In the context of combined antiretroviral therapy (cART), the boosted protease inhibitor (bPI) combination of darunavir with ritonavir (RTV) is frequently chosen as the preferred first- or second-line antiretroviral treatment, due to good efficacy, tolerability, and a high genetic barrier to resistance (1). There are some concerns with the use of these drugs, as they are prone to drug-drug interactions (DDIs), particularly with substrates or inducers of cytochrome P450 3A4 (CYP3A4) and P-glycoprotein (P-gp) (2–6). Concurrent use of rifampicin (RIF), a key component of combination antitubercular therapy, is expected to cause a significant reduction (about 57% for area under the concentration-time curve [AUC] and 80% for trough serum concentration [*C*_trough_] by *in silico* modeling [7]) in darunavir (DRV) with an 800/100 mg dose of ritonavir-boosted darunavir (DRV/r). This drug interaction is a major barrier to the use of DRV/r in countries where tuberculosis is endemic (7–9).

Recently, Ebrahim et al. reported that doubling the daily dose of DRV/r, in either one or two doses, did not adequately compensate DRV exposures in plasma for the induction by RIF, particularly with the *C*_trough_ (10). The same study showed an unacceptably high incidence of symptomatic hepatitis with marked alanine aminotransferase (ALT) elevations after the introduction of RIF to the DRV/r regimen, leading to premature closure of the study. Nevertheless, while the impact of this DDI on plasma concentrations of DRV was well described in this study, the effect of RIF on DRV drug disposition in circulating mononuclear cells is still unknown.

Since the concentrations of antiretrovirals (ARVs) within peripheral blood mononuclear cells (PBMCs) are more closely correlated with those in lymphoid tissues (11) than are plasma concentrations, intracellular PBMC concentrations provide useful information about antiretroviral disposition at the active sites. As the relative intracellular exposure of PIs can show significant intra- and interpatient variability, particularly considering different drug combinations, this information could explain variation in their antiviral effect (12–15). In this substudy, we aim to explore the concentrations of DRV, RTV, dolutegravir (DTG), and RIF within PBMCs in the context of the same prospective dose-escalation crossover study.

## RESULTS

### Patient characteristics and treatment safety.

A total of 17 participants were enrolled. All were of black African ethnicity, most were female (16, 94.2%), and their median (interquartile range [IQR]) age was 44 (39 to 47) years.

Median (IQR) body weight and body mass index (BMI) were 73 kg (67 to 91) and 31 kg/m^2^ (27 to 34), respectively; median (IQR) CD4 count at the baseline was 684 cell/mm^3^ (452 to 886), and median duration on their previous second-line ART was 58 months (IQR, 31 to 87). All patients were switching from an lopinavir-ritonavir (LPV/r)-based regimen and were virologically suppressed. In cohort 1, 1 of 5 patients developed symptomatic hepatic toxicity with grade 3 and 4 ALT elevation; 4 patients completed the full protocol. In cohort 2, 5 of 12 patients showed hepatic toxicity, and the study was stopped according to the stopping criteria. The hepatotoxicity developed 9 to 11 days after the introduction of RIF and 2 to 4 days after 100 mg RTV was added to DRV/r 800/100 mg once daily (QD).

### DRV *C*_trough_ and AUC in plasma and PBMCs.

**FIG 1:**
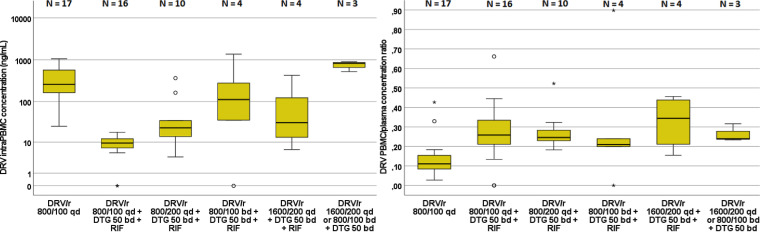
Distribution of the intra-PBMC DRV trough concentrations (left) and intra-PBMC/plasma concentration ratios (right) in each treatment period. Whiskers represents the first and fourth quartile, respectively; circles represent mild outliers (>2 standard deviations), and asterisks represent extreme outliers (>3 standard deviations).

After the addition of RIF, at the end of week 2, the concentrations of DRV measured in both plasma and PBMCs dropped significantly (*n* = 16; *P* < 0.001 by Wilcoxon test). The descriptive analysis of concentration data for each drug, with median values and IQR is summarized in Table S1 in the supplemental material. In patients who completed the full protocol, dose escalation did not sufficiently compensate for the inductive effect of RIF on DRV concentrations, which were significantly lower than before RIF addition (*n* = 4; *P* < 0.001 for both 800/100 mg twice daily [BD] and 1,600/200 mg QD by Wilcoxon test). DRV/RTV 800/100 BD resulted in slightly higher trough concentrations than 1,600/200 QD with RIF, although this difference did not reach statistical significance (*n* = 4; *P* = 0.101). Conversely, as summarized in Fig. 1, the differences within PBMCs were less pronounced. Differences in intra-PBMC DRV concentrations in 800/100 mg BD compared with 800/100 mg QD before RIF addition did not reach statistical significance (*n* = 4; *P* = 0.432), while the 1,600/100-mg QD dosage only resulted in a borderline reduction of intra-PBMC concentrations, both compared with 800/100 QD and 800/100 BD after RIF addition (*n* = 4; *P* = 0.068 for both differences). As depicted in Fig. 1, DRV PBMC/plasma concentration ratios were significantly increased (*n* = 16; *P* = 0.003) after the addition of RIF (week 2), and dose escalation did not result in statistically significant changes after week 2 (*n* = 10, 4, and 4, respectively; *P* > 0.324).

DRV area under the concentration-time curve from 0 to 24 h (AUC_0–24_) in plasma declined from a median value of 92,461 h · ng/mL (IQR, 39,655 to 197,735), before addition of RIF, to 45,532 h · ng/mL (IQR, 27,298 to 118,740; *P* = 0.114) and 34,695 h · ng/mL (IQR, 8,025 to 69,029; *P* = 0.068) with RIF and DRV/RTV dose escalation at 800/100 BD and 1,600/200 QD, respectively. AUC_0–24_ in PBMCs showed a similar reduction from a median (IQR) value of 17,503 h · ng/mL (10,975 to 34,876), before addition of RIF, to 11,100 h · ng/mL (8,756 to 31,100; *P* = 0.068) and 10,087 h · ng/mL (8,292 to 21,726; *P* = 0.068) with RIF and DRV/RTV dose escalation at 800/100 BD and 1,600/200 QD, respectively. A nonsignificant increase in the AUC PBMC/plasma ratio was observed for DRV after the addition of RIF and dose escalation to 800/100 BD and 1,600/200 QD (both *P* = 0.068) as summarized in Table S1.

### RTV *C*_trough_ and AUC in plasma and PBMCs.

As reported in Table S1, RTV trough plasma concentrations with the DRV/r 800/100-mg QD dose were significantly reduced after the addition of RIF (*P* < 0.001). DRV/RTV dose escalation to 800/100 mg BD resulted in RTV trough concentrations not significantly different to those before addition of RIF (*P* = 0.554), while trough concentrations on 1,600/200 mg QD were significantly lower (*P* < 0.001) than those without RIF on the 800/100-mg QD dose.

**FIG 2:**
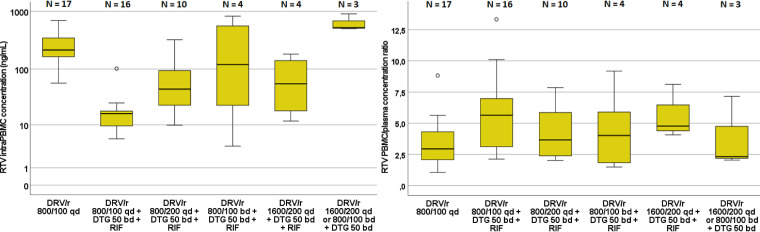
Distribution of the intra-PBMC RTV trough concentrations (left) and intra-PBMC/plasma trough concentration ratios (right) in each treatment period. Whiskers represents the first and fourth quartile, respectively; circles represent mild outliers (>2 standard deviations), and asterisks represent extreme outliers (>3 standard deviations).

Similar to what was observed for DRV, RTV trough concentrations in PBMCs appeared slightly less affected by the DDI than in plasma. RTV intra-PBMC concentrations were significantly reduced after the addition of RIF at week 2 (*P* < 0.001), while dose escalation to 800/100 BD yielded intra-PBMC concentrations not significantly lower than the ones before RIF addition (Fig. 2); conversely, the 1,600/200-mg QD regimen showed significantly lower intra-PBMC RTV through concentrations (*P* = 0.003). Compared with week 1, the intra-PBMC/plasma concentration ratio for RTV appeared significantly higher after the addition of RIF (week 2; *n* = 16; *P* = 0.012) and with the 1,600/200-mg QD dosage (*n* = 4; *P* = 0.045) as depicted in Fig. 2. Interestingly, the PBMC/plasma concentration ratio dropped again at week 6, after RIF withdrawal, suggesting that the observed increased ratio was actually due to the interaction with RIF.

### DTG *C*_trough_ and AUC in plasma and PBMCs.

**FIG 3:**
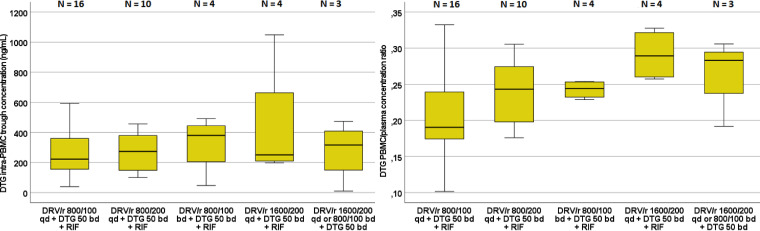
Distribution of the intra-PBMC DTG trough concentrations (left) and intra-PBMC/plasma trough concentration ratios (right) in each treatment period. Whiskers represents the first and fourth quartile, respectively; circles represent mild outliers (>2 standard deviations), and asterisks represent extreme outliers (>3 standard deviations).

Trough concentrations of DTG appeared mostly unchanged both in plasma and PBMCs among different treatment periods (Fig. 3: see also Table S2 in the supplemental material), confirming its robustness in terms of DDI profile.

Interestingly, significant differences in the intra-PBMC/plasma concentration ratio for DTG (*P* = 0.016 by Kruskal-Wallis test; *P* = 0.022 by Kendall test for paired samples) were observed between treatment periods, particularly when administered with DRV/RTV 1,600/200 QD dose. No significant differences were observed in terms of AUC_0–24_ in plasma and PBMCs (*P* = 0.785 and 0.773, respectively). A significant increasing trend was observed for DTG intra-PBMC/plasma ratio (*P* = 0.003) throughout the protocol, although no significant differences between treatment periods were confirmed by Kendal and Wilcoxon tests (also considering the low sample size), suggesting a progressive relative accumulation of DTG in PBMCs during this time and upon DRV/r dose escalation (Fig. 3).

### RIF *C*_max_ and AUC in plasma and PBMCs.

**FIG 4:**
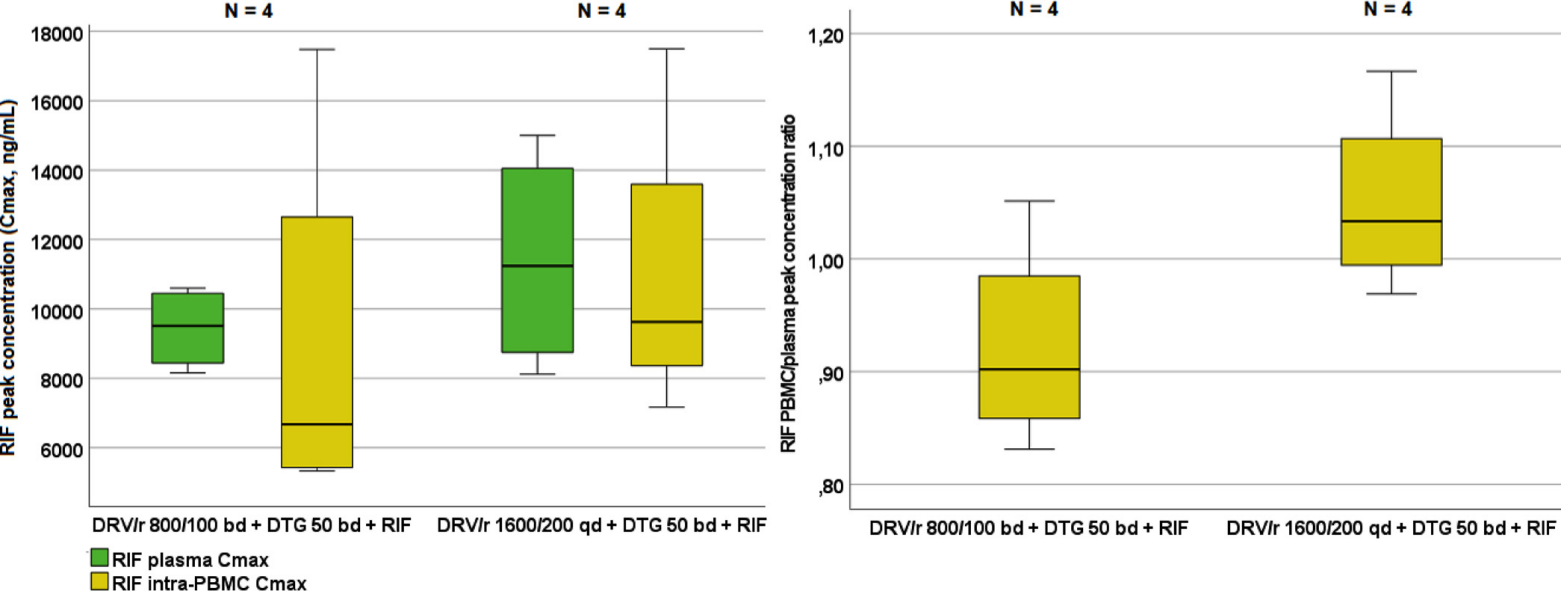
Distribution of the plasma and intra-PBMC RIF peak concentrations (left) and intra-PBMC/plasma peak concentration ratios (right) in each treatment period. Whiskers represents the first and fourth quartile, respectively; circles represent mild outliers (>2 standard deviations), and asterisks represent extreme outliers (>3 standard deviations).

As depicted in Fig. 4, no significant differences were observed between RIF maximum concentrations observed with DRV/RTV 800/100 BD and 1,600/200 QD doses, both in plasma and PBMCs (*P* = 0.465 and 0.144, respectively). No significant differences were observed in plasma or PBMCs in terms of RIF AUCs (*P* = 0.237 and 0.317, respectively) (see Table S2).

## DISCUSSION

To date, the magnitude of DDIs between strong cytochrome and P-gp inducers, such as RIF, and drugs which are substrates of these enzymes, as well as the appropriate eventual posological adjustments needed to overcome these DDIs, are commonly studied by evaluating variations in their plasma exposure. The impact of such DDIs on pharmacokinetic (PK) profiles could, however, vary significantly between plasma and intracellular compartments. The present study shows that DRV/r and DTG concentrations within PBMC after RIF addition and DRV/r dose escalation decreases in a slightly less marked way than in plasma, increasing the intra-PBMC/plasma ratio during the protocol.

The clinical relevance of this data cannot be overstated, as boosted protease inhibitors (bPIs), including DRV/r, remain the most commonly used ARVs in people living with HIV (PLWH) receiving second-line regimens in resource-limited settings. The DRV/r combination is known to be extremely prone to DDIs since both drugs are able to act both as perpetrators and victims.

RTV is both a strong inhibitor (3) and weak inducer of CYP3A4 and of P-gp (4, 5) and a substrate of these proteins, while DRV metabolism and elimination is strongly dependent by CYP3A4 and P-gp (2, 5, 6, 16, 17).

Recently, Ebrahim et al. (10) reported that doubling DRV/r dose once daily or twice daily did not compensate completely for the reduction in plasma exposure to DRV. Nevertheless, in the main, DRV plasma concentrations exceeded the 90% effective concentration (EC_90_) of 200 ng/mL for DRV (18) with the twice daily 800/100-mg dose, thus arguably sufficiently mitigating the DDI. Conversely, this study demonstrated an unacceptable high rate of hepatic toxicity to be the main problem associated with the administration of RIF and DRV plus adjusted doses of RTV. The mechanism underlying this toxicity remains poorly understood.

Through deepening the PK evaluation in these patients to the intracellular level, the present study highlighted that the magnitude of the changes in drug concentrations in plasma and PBMCs were significantly different, with an increase in the intra-PBMC/plasma concentration ratio after the addition of RIF for both DRV and RTV. This evidence could impact both treatment efficacy and safety. In weighing the impact of the DDI on antiviral activity, the relative increase in the intracellular disposition for DRV and RTV should be considered. Therefore, while PK data in plasma and PBMC would suggest that DRV/r dose escalation could be effective from a virological point of view, the high rate of hepatic toxicity indicates that this option cannot be recommended. Regarding the possible explanations for this toxic effect and in light of the timing of its occurrence and of the observed relative increase in the intracellular disposition of DRV/r, we could suppose an increased concentration of these drugs (all characterized by a mild hepatotoxic potential [18–21]) within hepatocytes, in synergy with a significant alteration in their intracellular metabolism and a potential “trapping” effect due to the presence of RTV.

Concerning the impact of altered metabolism on drug toxicity, a recent work showed that activation of the pregnane X receptor (PXR) by RIF or efavirenz increased the hepatotoxic potential of RTV in a transgenic mouse model. This effect, clearly mediated by heightened CYP3A4 function and indicated by RTV intracellular metabolites (particularly M1 and M13), was most likely responsible for the observed toxicity (19).

Conversely, previous data showed that LPV/r dose escalation was effective in overcoming RIF induction and relatively well tolerated (22). Nevertheless, RTV intracellular exposure in the presence of DRV was previously reported to be slightly higher than that with LPV (14, 15). Therefore, the combined effect of higher intracellular RTV penetration and higher production of hepatotoxic metabolites (19-fold increase for M1 and 9-fold increase for M13 in the mouse model) might explain the observed phenomenon. Alternatively, *in vitro* data discourage the use of cobicistat as a safer alternative booster due to lower boosting effect on DRV, particularly with RIF. Finally, based on the observed PK data, we found no evidence to support safety concerns due to the RIF and DTG concentrations. RIF exposure in terms of maximum concentration of drug in serum (*C*_max_) and AUC, both in plasma and PBMCs, appeared comparable with previous data: the observed similar RIF concentrations in plasma and PBMC in accordance with previous works (23) suggest that the antitubercular activity of RIF would be maintained in the presence of DRV/r and DTG. Similarly, DTG *C*_trough_ in PBMC appeared lower than in plasma (about 25%) throughout the protocol in accordance with previous data (24). Actually, a slight increase appeared in RIF *C*_max_ in 3 patients out of 4, with DRV/r 1,600/200 QD, maybe due to the capability of RTV to inhibit OATP1B1 and P-gP, increasing RIF bioavailability (25–27). Nevertheless, the low sample size does not allow us to draw definitive conclusions. Conversely, by a technical point of view, drug loss from PBMCs during cell washing could have affected drug quantification to some extent despite the possible magnitude for this possible bias still being debated (from 10% to 50% according to previous works [28–30]); nevertheless, since all of the PBMC samples were processed following the same protocol, any possible bias was normalized in the comparisons among treatment periods. Taken together, the evidence from this study indicates the intracellular PK evaluation as a useful tool to better understand the results from clinical trials concerning DDIs, confirming or not their theoretical relevance and/or the appropriateness of dose modification strategies.

## MATERIALS AND METHODS

### Study design and patient enrollment.

PLWH on cART with boosted PIs and dual nucleoside reverse transcriptase inhibitors (NRTIs) were enrolled into the “DaRifi” study (ClinicalTrials registration no. NCT03892161) approved by the University of Cape Town Human Research Ethics Committee and the South African Health Products Regulatory Authority. All patients signed informed consent before enrollment, in compliance with the Declaration of Helsinki and the standards of good clinical practice.

The study was an open-label, single-center, dose-escalation crossover study to determine the pharmacokinetics (PK) of DRV, RTV, and DTG in the presence or absence of RIF. The planned enrollment of 28 participants was scheduled in 3 consecutive cohorts (cohort 1, 5 patients; cohort 2, 11 patients; cohort 3, 12 patients) in order to allow early study discontinuation in case of major safety concerns; stopping criteria for the study were 2 out of 5 patients experiencing serious adverse events (SAE) in the first cohort or a cumulative incidence of SAE higher than 20% with the following cohorts. Review of the safety data from the first cohort by the independent data and safety monitoring committee was completed before starting enrollment into cohort 2. Patients were recruited from the antiretroviral clinic of the Hannan Crusaid Treatment Centre in Gugulethu (Cape Town, South Africa). Patients were aged between 18 and 60 years, virologically suppressed (HIV-1 RNA < 50 copies/mL) for at least 3 months, and with a CD4 count higher than 200 cells/mL. Exclusion criteria included the following: active or suspected tuberculosis (TB), impaired hepatic function or documented cirrhosis, hepatitis C virus (HCV) or hepatitis B virus (HBV) coinfection, ALT elevation > 2.5 times the upper limit of normal, grade 3 or 4 hematological abnormalities, AIDS-defined illness, and impaired renal function (estimated glomerular filtration rate < 50 mL/min, calculated through the MDRD formula). The study protocol was based on 6 different treatment periods of 1 week each, with a total study duration of 6 weeks for each patient. Patients started the study (week 1) switching their previous PI to the standard DRV/r dose of 800/100 mg once daily (QD), continuing their NRTI backbone. Then, DTG 50 mg twice daily (BD) and RIF 600 or 750 mg QD for volunteers weighing less or more than 70 kg, respectively, were added (week 2) at day 7. At day 14, RTV was increased to 200 mg QD (week 3), and subsequently, at day 21, patients were randomized in a 1:1 proportion to receive either 800/100 mg BD during week 4 and then switched to 1,600/200 mg QD during week 5 or vice versa. Finally, RIF was withdrawn while DRV/r dose was left unchanged during week 6.

### Drug measurement in plasma and PBMCs.

Blood sampling for PK purposes was carried out using a 4-mL EDTA tube for each plasma time point, and 2 cell preparation tubes (CPT) (Becton, Dickinson and Co., Franklin Lakes, NJ, USA) for the isolation of PBMCs. The intensive PK sampling for the definition of the area under the concentration effect curve (AUC) was based on sampling before dose (*C*_trough_) and at 0.5, 1, 2, 4, 6, 8, 12, and 24 h after an observed dose. The measurement of DRV, RTV, and RIF concentrations in plasma was performed at the University of Cape Town through different liquid chromatography-tandem mass spectrometry (LC-MS/MS) methods previously described (10, 23, 31). DTG was analyzed with a validated LC-MS/MS method developed at the Division of Clinical Pharmacology, University of Cape Town. Briefly, samples were processed with a liquid-liquid extraction method using dolutegravir-d4 as the internal standard, followed by high-pressure liquid chromatography-tandem mass spectrometry (HPLC-MS/MS) analysis using an AB SCIEX API 4000 instrument. The analyte and internal standard were monitored at mass transitions of the protonated precursor ions *m/z* 420.1 and *m/z* 424.2 to the product ions *m/z* 277.2 and *m/z* 279.1, respectively. The calibration curve fitted a quadratic regression model over the range of 0.030 to 10.0 μg/mL. Accuracy (%bias) and precision (% coefficient of variation [CV]) statistics of the quality control samples were between 103.5% and 106.0% and 4.6% and 6.1%, respectively. The laboratory participated in the Clinical Pharmacology Quality Assurance external quality control program under a contract with the Division of AIDS of the National Institute of Allergy and Infectious Diseases. The assay was Clinical Pharmacology Quality Assurance program (CPQA) approved.

Simplified PK sampling was performed for PBMC determinations, including sampling before dose intake and 2 and 6 h after dose. In order to accurately determine the capability of dose escalation to compensate for metabolic induction, intensive PK sampling was scheduled at day 7 (at the steady-state for DRV/r, before the addition of RIF), day 28, and day 35, describing the plasma and PBMC PK at the theoretically steady-state of DRV/r 800/100 mg BD and 1,600/200 mg QD treatment periods. A single predose sampling was also performed at the end of each other treatment period to determine the steady-state trough concentrations. The evaluation of RIF concentrations was performed only at weeks 4 and 5, since RIF trough concentrations are expected to be undetectable and since its antimicrobial activity is led by its *C*_max_. PBMCs were isolated from blood collected with cell preparation tubes (CPT vacutainer; Becton, Dickinson, Franklin Lakes, NJ, USA) based on a separation by density gradient through centrifugation and 2 washing steps as previously described in several works (24, 32, 33); the cell pellets were lysed with water/methanol 30:70 and then shipped on dry ice to the Laboratory of Clinical Pharmacology and Pharmacokinetics of the University of Turin where the analyses were performed.

The quantifications of DRV, RTV, DTG, and RIF in PBMCs were performed through the application of two previously published validated ultra-high performance liquid chromatography coupled to tandem mass spectrometry (UHPLC-MS/MS) methods for the quantification of ARVs (32) and anti-TB drugs (24), respectively. Cell number in each sample and the mean cellular volume for PBMCs was used to normalize the drug amounts within PBMCs, obtaining concentrations expressed in nanograms per milliliter. A turbidimetric assay was performed using an HPLC system equipped with a photodiode array (PDA) detector, settled in fast injection analysis (without column) and monitoring light absorption at 260 nm (the most specific one for DNA). The assay was calibrated with a PBMC aliquot of known cell number in a range between 1 × 10^6^ and 4 × 10^7^ cells/mL and isolated with the same protocol used for patient samples from buffy coats supplied by the blood bank of the “Città della Salute e della Scienza” of Turin. A cross-validation was performed between the standard automated count through a Z2 cell counter (Beckman Coulter, Milan, Italy) and this turbidimetric count on 30 samples extracted from buffy-coats, showing a mean between-assay bias of 6.3% (relative standard deviation of 6.7%). A mean corpuscular volume of 282.9 fl was considered to calculate the total volume of cells in order to obtain intracellular concentrations, as described by Simiele et al. (33).

### Statistical analysis.

Derivation of the plasma PK measures was performed using noncompartmental analysis. The intra-PBMC AUC_0–24_ values of DRV, RTV, DTG, and RIF were calculated through Phoenix WinNonlin software (version 8.1; Certara, Princeton, NJ, USA). For the twice-daily regimens, AUC_0–24_ was calculated by doubling AUC_0-12_. A “linear-up/log-down” algorithm was applied for the integration of simplified AUC data (0, 2, 6, and 12 h after dose) in order to avoid overestimation. An adjusted determination coefficient (*R*^2^) for the calculation of the predicted/observed AUCs of at least 0.8 was considered as the minimum acceptable cutoff. All AUC data and trough concentrations were evaluated on the last day of each treatment period at the theoretical steady state; *C*_trough_ were considered as the *C*_0_. The concentration at 2 h was used as an approximation of the *C*_max_ for the intra-PBMC PK. Drugs concentrations in plasma and PBMC were described as median values and interquartile ranges (IQR), considering the low sample size and the difficulty to assess distribution normality. Differences between treatment periods were evaluated through nonparametric tests for independent samples (Kruskal-Wallis and Mann-Whitney tests) or for patients who completed the full protocol for coupled samples (Kendall and Wilcoxon tests).

## References

[1] Lefebvre E, Schiffer CA. 2008. Resilience to resistance of HIV-1 protease inhibitors: profile of darunavir. AIDS Rev 10:131–142. 18820715 PMC2699666

[2] Brown KC, Paul S, Kashuba AD. 2009. Drug interactions with new and investigational antiretrovirals. Clin Pharmacokinet 48:211–241. 10.2165/00003088-200948040-00001. 19492868 PMC2857544

[3] Drewe J, Gutmann H, Fricker G, Torok M, Beglinger C, Huwyler J. 1999. HIV protease inhibitor ritonavir: a more potent inhibitor of P-glycoprotein than the cyclosporine analog SDZ PSC 833. Biochem Pharmacol 57:1147–1152. 10.1016/S0006-2952(99)00026-X. 11230802

[4] Perloff MD, Von Moltke LL, Marchand JE, Greenblatt DJ. 2001. Ritonavir induces P-glycoprotein expression, multidrug resistance-associated protein (MRP1) expression, and drug transporter-mediated activity in a human intestinal cell line. J Pharm Sci 90:1829–1837. 10.1002/jps.1133. 11745741

[5] Rehman S, Nabi B, Fazil M, Khan S, Bari NK, Singh R, Ahmad S, Kumar V, Baboota S, Ali J. 2017. Role of P-glycoprotein inhibitors in the bioavailability enhancement of solid dispersion of darunavir. Biomed Res Int 2017:8274927. 10.1155/2017/8274927. 29226149 PMC5684613

[6] Rittweger M, Arasteh K. 2007. Clinical pharmacokinetics of darunavir. Clin Pharmacokinet 46:739–756. 10.2165/00003088-200746090-00002. 17713972

[7] Lutz JD, Kirby BJ, Wang L, Song Q, Ling J, Massetto B, Worth A, Kearney BP, Mathias A. 2018. Cytochrome P450 3A induction predicts P-glycoprotein induction; part 1: establishing induction relationships using ascending dose rifampin. Clin Pharmacol Ther 104:1182–1190. 10.1002/cpt.1073. 29569723 PMC6282691

[8] Lutz JD, Kirby BJ, Wang L, Song Q, Ling J, Massetto B, Worth A, Kearney BP, Mathias A. 2018. Cytochrome P450 3A induction predicts P-glycoprotein induction; part 2: prediction of decreased substrate exposure after rifabutin or carbamazepine. Clin Pharmacol Ther 104:1191–1198. 10.1002/cpt.1072. 29569712 PMC6282692

[9] Roberts O, Khoo S, Owen A, Siccardi M. 2017. Interaction of rifampin and darunavir-ritonavir or darunavir-cobicistat in vitro. Antimicrob Agents Chemother 61:e01776-16. 10.1128/AAC.01776-16. 28193650 PMC5404587

[10] Ebrahim I, Maartens G, Wiesner L, Orrell C, Smythe W, McIlleron H. 2020. Pharmacokinetic profile and safety of adjusted doses of darunavir/ritonavir with rifampicin in people living with HIV. J Antimicrob Chemother 75:1019–1025. 10.1093/jac/dkz522. 31942627 PMC8453380

[11] Dyavar SR, Gautam N, Podany AT, Winchester LC, Weinhold JA, Mykris TM, Campbell KM, Alnouti Y, Fletcher CV. 2019. Assessing the lymphoid tissue bioavailability of antiretrovirals in human primary lymphoid endothelial cells and in mice. J Antimicrob Chemother 74:2974–2978. 10.1093/jac/dkz273. 31335938 PMC6753470

[12] Fletcher CV, Staskus K, Wietgrefe SW, Rothenberger M, Reilly C, Chipman JG, Beilman GJ, Khoruts A, Thorkelson A, Schmidt TE, Anderson J, Perkey K, Stevenson M, Perelson AS, Douek DC, Haase AT, Schacker TW. 2014. Persistent HIV-1 replication is associated with lower antiretroviral drug concentrations in lymphatic tissues. Proc Natl Acad Sci USA 111:2307–2312. 10.1073/pnas.1318249111. 24469825 PMC3926074

[13] Mitchell C, Roemer E, Nkwopara E, Robbins B, Cory T, Rue T, Fletcher CV, Frenkel L. 2014. Correlation between plasma, intracellular, and cervical tissue levels of raltegravir at steady-state dosing in healthy women. Antimicrob Agents Chemother 58:3360–3365. 10.1128/AAC.02757-13. 24687503 PMC4068472

[14] D'Avolio A, Simiele M, Calcagno A, Siccardi M, Larovere G, Agati S, Baietto L, Cusato J, Tettoni M, Sciandra M, Trentini L, Di Perri G, Bonora S. 2013. Intracellular accumulation of ritonavir combined with different protease inhibitors and correlations between concentrations in plasma and peripheral blood mononuclear cells. J Antimicrob Chemother 68:907–910. 10.1093/jac/dks484. 23221630

[15] De Nicolò A, Simiele M, Calcagno A, Abdi AM, Bonora S, Di Perri G, D'Avolio A. 2014. Intracellular antiviral activity of low-dose ritonavir in boosted protease inhibitor regimens. Antimicrob Agents Chemother 58:4042–4047. 10.1128/AAC.00104-14. 24798279 PMC4068548

[16] Arab-Alameddine M, Lubomirov R, Fayet-Mello A, Aouri M, Rotger M, Buclin T, Widmer N, Gatri M, Ledergerber B, Rentsch K, Cavassini M, Panchaud A, Guidi M, Telenti A, Decosterd LA, Csajka C, Swiss HIV Cohort Study. 2014. Population pharmacokinetic modelling and evaluation of different dosage regimens for darunavir and ritonavir in HIV-infected individuals. J Antimicrob Chemother 69:2489–2498. 10.1093/jac/dku131. 24821595

[17] Konig SK, Herzog M, Theile D, Zembruski N, Haefeli WE, Weiss J. 2010. Impact of drug transporters on cellular resistance towards saquinavir and darunavir. J Antimicrob Chemother 65:2319–2328. 10.1093/jac/dkq324. 20817741

[18] Tibotec, Inc. 2008. Prezista. Tibotec, Inc., Yardley, PA. https://www.accessdata.fda.gov/drugsatfda_docs/label/2008/021976s003s004lbl.pdf.

[19] Shehu AI, Lu J, Wang P, Zhu J, Wang Y, Yang D, McMahon D, Xie W, Gonzalez FJ, Ma X. 2019. Pregnane X receptor activation potentiates ritonavir hepatotoxicity. J Clin Invest 129:2898–2903. 10.1172/JCI128274. 31039134 PMC6597219

[20] Sulkowski MS. 2003. Hepatotoxicity associated with antiretroviral therapy containing HIV-1 protease inhibitors. Semin Liver Dis 23:183–194. 10.1055/s-2003-39949. 12800071

[21] Sulkowski MS, Thomas DL, Chaisson RE, Moore RD. 2000. Hepatotoxicity associated with antiretroviral therapy in adults infected with human immunodeficiency virus and the role of hepatitis C or B virus infection. JAMA 283:74–80. 10.1001/jama.283.1.74. 10632283

[22] Decloedt EH, McIlleron H, Smith P, Merry C, Orrell C, Maartens G. 2011. Pharmacokinetics of lopinavir in HIV-infected adults receiving rifampin with adjusted doses of lopinavir-ritonavir tablets. Antimicrob Agents Chemother 55:3195–3200. 10.1128/AAC.01598-10. 21537021 PMC3122443

[23] McIlleron H, Hundt H, Smythe W, Bekker A, Winckler J, van der Laan L, Smith P, Zar HJ, Hesseling AC, Maartens G, Wiesner L, van Rie A. 2016. Bioavailability of two licensed paediatric rifampicin suspensions: implications for quality control programmes. Int J Tuber Lung Dis 20:915–919. 10.5588/ijtld.15.0833. PMC4978631 27287644

[24] Baietto L, Calcagno A, Motta I, Baruffi K, Poretti V, Di Perri G, Bonora S, D'Avolio A. 2015. A UPLC-MS-MS method for the simultaneous quantification of first-line antituberculars in plasma and in PBMCs. J Antimicrob Chemother 70:2572–2575. 10.1093/jac/dkv148. 26066583

[25] Asaumi R, Toshimoto K, Tobe Y, Hashizume K, Nunoya KI, Imawaka H, Lee W, Sugiyama Y. 2018. Comprehensive PBPK model of rifampicin for quantitative prediction of complex drug-drug interactions: CYP3A/2C9 induction and OATP inhibition effects. CPT Pharmacometrics Syst Pharmacol 7:186–196. 10.1002/psp4.12275. 29368402 PMC5869557

[26] Rockwood N, Meintjes G, Chirehwa M, Wiesner L, McIlleron H, Wilkinson RJ, Denti P. 2016. HIV-1 coinfection does not reduce exposure to rifampin, isoniazid, and pyrazinamide in South African tuberculosis outpatients. Antimicrob Agents Chemother 60:6050–6059. 10.1128/AAC.00480-16. 27480859 PMC5038257

[27] Shitara Y, Takeuchi K, Horie T. 2013. Long-lasting inhibitory effects of saquinavir and ritonavir on OATP1B1-mediated uptake. J Pharm Sci 102:3427–3435. 10.1002/jps.23477. 23440887

[28] Cory TJ, Winchester LC, Robbins BL, Fletcher CV. 2015. A rapid spin through oil results in higher cell-associated concentrations of antiretrovirals compared with conventional cell washing. Bioanalysis 7:1447–1455. 10.4155/bio.15.70. 26168252 PMC4512735

[29] Ford J, Boffito M, Wildfire A, Hill A, Back D, Khoo S, Nelson M, Moyle G, Gazzard B, Pozniak A. 2004. Intracellular and plasma pharmacokinetics of saquinavir-ritonavir, administered at 1,600/100 milligrams once daily in human immunodeficiency virus-infected patients. Antimicrob Agents Chemother 48:2388–2393. 10.1128/AAC.48.7.2388-2393.2004. 15215085 PMC434222

[30] Khoo SH, Hoggard PG, Williams I, Meaden ER, Newton P, Wilkins EG, Smith A, Tjia JF, Lloyd J, Jones K, Beeching N, Carey P, Peters B, Back DJ. 2002. Intracellular accumulation of human immunodeficiency virus protease inhibitors. Antimicrob Agents Chemother 46:3228–3235. 10.1128/AAC.46.10.3228-3235.2002. 12234849 PMC128776

[31] Rabie H, Rawizza H, Zuidewind P, Winckler J, Zar H, Van Rie A, Wiesner L, McIlleron H. 2019. Pharmacokinetics of adjusted-dose 8-hourly lopinavir/ritonavir in HIV-infected children co-treated with rifampicin. J Antimicrob Chemother 74:2347–2351. 10.1093/jac/dkz171. 31081020 PMC6640304

[32] De Nicolò A, Ianniello A, Ferrara M, Avataneo V, Cusato J, Antonucci M, De Vivo E, Waitt C, Calcagno A, Trentalange A, Muccioli G, Bonora S, Di Perri G, D'Avolio A. 2021. Validation of a UHPLC-MS/MS method to quantify twelve antiretroviral drugs within peripheral blood mononuclear cells from people living with HIV. Pharmaceuticals (Basel) 14:12. 10.3390/ph14010012. PMC7824452 33375547

[33] Simiele M, D'Avolio A, Baietto L, Siccardi M, Sciandra M, Agati S, Cusato J, Bonora S, Di Perri G. 2011. Evaluation of the mean corpuscular volume of peripheral blood mononuclear cells of HIV patients by a coulter counter to determine intracellular drug concentrations. Antimicrob Agents Chemother 55:2976–2978. 10.1128/AAC.01236-10. 21402849 PMC3101420

