# Supplementary material for: The Effect of Rifampicin on Darunavir, Ritonavir, and Dolutegravir Exposure within Peripheral Blood Mononuclear Cells: a Dose Escalation Study
Source: Antimicrob Agents Chemother. 2022 May 18;66(6):e00136-22. doi: 10.1128/aac.00136-22 (PMC9211429; doi:10.1128/aac.00136-22)
Supplement: Supplemental file 1 — Tables S1 and S2. Download aac.00136-22-s0001.pdf, PDF file, 0.1 MB [file aac.00136-22-s0001.pdf]

Supplementary Table 1: Summary of PK data in plasma and PBMC for DRV and RTV (C_{trough} data are described only for DRV) at the end of each treatment period (steady-state); data are shown as median values (interquartile ranges). N.a. = not applicable; n.d. = not determinable; n.c. = not computable.

	**DRV/RTV 800/100 QD (n = 17)**	**DRV/RTV 800/100 QD + RIF +DTG (n = 16)**	**DRV/RTV 800/200 QD + RIF +DTG (n = 10)**	**DRV/RTV 800/100 BD + RIF + DTG (n = 4)**	**DRV/RTV 1600/200 QD + DTG + RIF (n = 4)**	**Last DRV/r regimen + DTG without RIF (n = 3)**
**DRV C_{trough} plasma (ng/mL)**	2370 (839 – 7135)	37 (19 – 52)	86 (45 – 245)	536 (27 – 3540)	126 (31 – 1244)	3550 (1660 – n.c.)
**DRV C_{trough} PBMC (ng/mL)**	261 (158 – 577)	10 (n.d. – 13)	25 (12 – 67)	112 (18 – 820)	31 (12 – 331)	830 (525 – n.c.)
**DRV PBMC/Plasma C_{trough} ratio**	0.11 (0.08 – 0.16)	0.26 (0.21 – 0.34)	0.25 (0.23 – 0.29)	0.21 (0.10 – 0.57)	0.34 (0.18 – 0.45)	0.24 (0.23 - n.c.)
**DRV AUC_{0-24} Plasma (h*ng/mL)**	92461 (39655 – 197735)	n.a.	n.a.	45532 (27298 – 118740)	34695 (8025 – 69029)	n.a.
**DRV AUC_{0-24} PBMC (h*ng/mL)**	17503 (10975 – 34876)	n.a.	n.a.	11100 (8756 – 31100)	10087 (8292 – 21726)	n.a.
**RTV C_{trough} plasma (ng/mL)**	77.5 (57.2 – 97.6)	2.5 (2.5 – 2.5)	11.9 (5.2 – 23.8)	65.2 (2.5 – 150.5)	10.1 (3.6 – 31.7)	216.0 (72.9 - n.c.)
**RTV C_{trough} PBMC (ng/mL)**	215.1 (144.2 – 374.2)	16.2 (9.4 – 18.0)	44.6 (20.2 – 95.6)	120.0 (13.3 – 694.2)	68.4 (15.8 – 163.9)	521.4 (504.3 - n.c.)
**RTV PBMC/Plasma C_{trough} ratio**	2.95 (2.01 – 4.39)	5.64 (2.96 – 7.09)	3.67 (2.34 – 6.20)	4.02 (1.66 – 7.54)	4.77 (4.23 – 7.29)	2.33 (2.05 - n.c.)
**RTV AUC_{0-24} Plasma (h*ng/mL)**	6069 (3635 – 7853)	n.a.	n.a.	6642 (2098 – 9779)	6517 (2938 – 8996)	n.a.
**RTV AUC_{0-24} PBMC (h*ng/mL)**	9829 (8550 – 12410)	n.a.	n.a.	13606 (6444 – 21352)	14475 (7179 – 18793)	n.a.

Supplementary Table 2: Summary of PK data in plasma and PBMC for DTG and RIF (C_{trough} data are described only for DTG) at the end of each treatment period (steady-state); data are shown as median values (interquartile ranges). N.a. = not applicable; n.d. = not determinable; n.c. = not computable.

	**DRV/RTV 800/100 QD (n = 17)**	**DRV/RTV 800/100 QD + RIF +DTG (n = 16)**	**DRV/RTV 800/200 QD + RIF +DTG (n = 10)**	**DRV/RTV 800/100 BD + RIF + DTG (n = 4)**	**DRV/RTV 1600/200 QD + DTG + RIF (n = 4)**	**Last DRV/r regimen + DTG without RIF (n = 3)**
**DTG C_{trough} Plasma (ng/mL)**	n.a.	1245 (757 – 1832)	1248 (524 – 1727)	1431 (120 – 1911)	962 (669 – 2758)	1382 (1215 – n.c.)
**DTG C_{trough} PBMC (ng/mL)**	n a.	223 (152 – 378)	274 (145 – 389)	363 (29 – 444)	251 (204 – 856)	344 (290 – n.c.)
**DTG PBMC/Plasma C_{trough} ratio**	n.a.	0.19 (0.17 – 0.24)	0.24 (0.19 – 0.28)	0.24 (0.210 – 0.253)	0.29 (0.26 – 0.33)	0.28 (0.27 - n.c.)
**DTG AUC_{0-24} Plasma (h*ng/mL)**	n.a.	n.a.	n.a.	31646 (23015 – 37240)	30967 (21915 – 41292)	n.a.
**DTG AUC_{0-24} PBMC (h*ng/mL)**	n.a.	n.a.	n.a.	14889 (10813 – 17636)	12807 (11364 – 24391)	n.a.
**RIF C_{max} Plasma (ng/mL)**	n.a.	n.a.	n.a.	9515 (8303 – 10525)	11240 (8435 – 14525)	n.a.
**RIF C_{max} PBMC (ng/mL)**	n.a.	n.a.	n.a.	6675 (5384 – 10314)	9630 (7771 – 15546)	n.a.
**RIF PBMC/Plasma C_{max} ratio**	n.a.	n.a.	n.a.	0.90 (0.85 – 1.02)	1.03 (0.98 – 1.14)	n.a.
**RIF AUC_{0-24} Plasma (h*ng/mL)**	n.a.	n.a.	n.a.	48802 (41998 – 60339)	53159 (46596 – 69438)	n.a.
**RIF AUC_{0-24} PBMC (h*ng/mL)**	n.a.	n.a.	n.a.	59327 (53482 – 90796)	67443 (39849 – 110052)	n.a.
